# A 26‐Year Record of Seasonality and Interannual Variability in Marine Mammal Sightings From Northern Marguerite Bay, Antarctica

**DOI:** 10.1002/ece3.73317

**Published:** 2026-03-23

**Authors:** Andrew Clarke, Alysa Fisher, Hugh J. Venables, Lucy Allen, Richard G. Davies

**Affiliations:** ^1^ British Antarctic Survey Cambridge UK; ^2^ School of Environmental Sciences University of East Anglia Norwich UK; ^3^ School of Biological Sciences University of East Anglia Norwich UK

**Keywords:** Antarctic, climate change, ice, seal, seasonality, whale

## Abstract

Whales and seals are an important and often conspicuous component of the Southern Ocean pelagic fauna. Some species have been studied extensively, but almost exclusively in the austral summer, and winter data for any species are sparse. Here we report the results from a 26‐year record (1998–2023) of year‐round weekly observations of marine mammals in Ryder Bay, an inlet of northern Marguerite Bay on the western Antarctic Peninsula. Humpback Whales are summer visitors and can be present any time between December and May, though a few individuals may remain over winter. Minke Whales and Killer Whales are present year‐round, as are the three ice‐obligate phocid seals, Crabeater, Weddell and Leopard Seals. A few (1–12) Weddell Seal pups are born in most years. Southern Elephant Seals are seasonal visitors, with variable numbers hauled out to moult from November to June; a small number (1–6) of pups are born occasionally. Antarctic Fur Seals are present from February to June, often in large numbers, typically leaving once fast ice starts to form. All species were seen in pack‐ice concentrations ranging from open water to close pack, but none were seen once extensive fast ice developed. In those species that were present year‐round, sighting frequency was lower in winter than summer, suggesting a seasonal movement away from nearshore waters. Sighting frequency of Humpback Whales has increased over the study period, with whales arriving earlier by ~18 days per decade, and staying longer. Over the same period, sightings of Minke Whales have become less frequent, and those of Killer Whales more frequent. These changes are likely linked to shifts in ice dynamics driven by regional climate change along the western Antarctic Peninsula.

## Graphical abstract

Whales and seals are an important and often conspicuous component of the Southern Ocean pelagic fauna but data from winter are very sparse. Six species remain in Marguerite Bay, Antarctica, year‐round while three are seasonal visitors. Humpback Whales are summer visitors and show a long‐term increase in sighting frequency together with an earlier arrival and a longer stay.

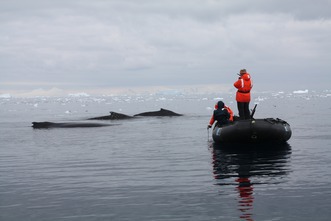

## 1 Introduction

The Southern Ocean supports a diverse assemblage of marine mammals where they form an important and often conspicuous component of the pelagic fauna. The larger cetaceans were heavily exploited in the early twentieth century and while this led to the near extinction of some species, biologists working at South Georgia for the *Discovery* Investigations were able to document the basic biology of many of the target species (Hardy 1967). Shore‐based whaling ceased with closure of the last whaling station on South Georgia in 1966, but the subsequent expansion of scientific research in Antarctica has allowed research on marine mammals to shift in focus and concentrate on what can be learned from individuals in the wild rather than a harvested carcass. Some species have been relatively well studied, particularly Humpback Whale and Killer Whale. In contrast, Antarctic Minke Whale remains among the least known of species, and others such as Arnoux's Beaked Whale and Ross Seal are encountered only rarely.

In the Atlantic and Pacific sectors of the Southern Ocean, most observations come from South Georgia or the northern Antarctic Peninsula, and from summer. By contrast, the marine mammal fauna of the more southerly Marguerite Bay is poorly known, mainly because until recently extensive winter fast‐ice and frequent dense summer pack‐ice meant that it was often difficult for ships to penetrate the bay, especially early in the summer season. There have been few scientific cruises in the area and there are only three scientific stations that are occupied year‐round. As with much of Antarctica, biological observations in winter are scarce. Over the period of this study scientific cruises visited Marguerite Bay during winter only in 2001 and 2002 (Thiele et al. 2004; Friedlaender et al. 2006) and furthermore not all of these were able to enter deep into the bay.

The western Antarctic Peninsula is experiencing rapid regional climate change: air temperature is rising, the nature and timing of precipitation is changing, and sea‐ice dynamics are shifting (Clarke et al. 2007; Stammerjohn et al. 2008, 2015; Ducklow et al. 2013). These changes are particularly striking in the more northerly sections of the Antarctic Peninsula but shifts in sea‐ice dynamics are evident in Marguerite Bay and round into the Bellingshausen and Amundsen Seas (Himmich et al. 2024), and in June 2025 RRS *Sir David Attenborough* encountered extensive areas of ice‐free water in northern Marguerite Bay.

The annual advance and retreat of sea‐ice is one of the largest seasonal changes on the planet, and is of profound significance for the marine ecosystem as the ecology of many polar marine mammals is tied intimately to sea‐ice. The change in sea‐ice dynamics associated with the regional climate change evident along the western Antarctic Peninsula is thus likely to be having impacts on the marine ecosystem (Clarke et al. 2007; Ducklow et al. 2012, 2013). These impacts may be particularly evident at higher levels of the food‐web, which have the potential to integrate changes at lower levels. The potential for climate change in polar regions to affect marine mammals has long been recognised (Tynan and DeMaster 1997) and while critical habitat for breeding can be identified relatively easily for seals and some coastal cetaceans, identifying critical habitat for foraging is more difficult, especially in pelagic species (Harwood 2001). Gulland et al. (2022) reviewed predicted impacts of climate change for Arctic marine mammals and found that for seven species, specific predictions were not yet possible. These species included Humpback Whale and Killer Whale.

The top predators in the Southern Ocean include both seabirds and marine mammals. Changes in the distribution and breeding phenology of penguins along the western Antarctic Peninsula have been well documented (Ducklow et al. 2013; Martinez et al. 2026), but much less is known of impacts on marine mammals, although changes in the arrival time of Humpback Whales have been suggested (Cimino et al. 2023).

Ecology typically moves from the establishment of patterns to the exploration of the processes that underpin them (Lawton 1999). Patterns can be spatial or temporal, and one of the boldest temporal patterns is seasonality. The aim of this study was to document the seasonal occurrence of marine mammals in northern Marguerite Bay from land‐based observations undertaken year‐round, with particular emphasis on the relationship between marine mammal presence and the different forms of sea‐ice, the quantification of interannual variability and, in those species present only seasonally, the detection of any phenological changes such as shifts in arrival or departure dates and relating these where possible to changes in sea‐ice dynamics.

## 2 Methods

### 2.1 Study Area

**FIGURE 1:**
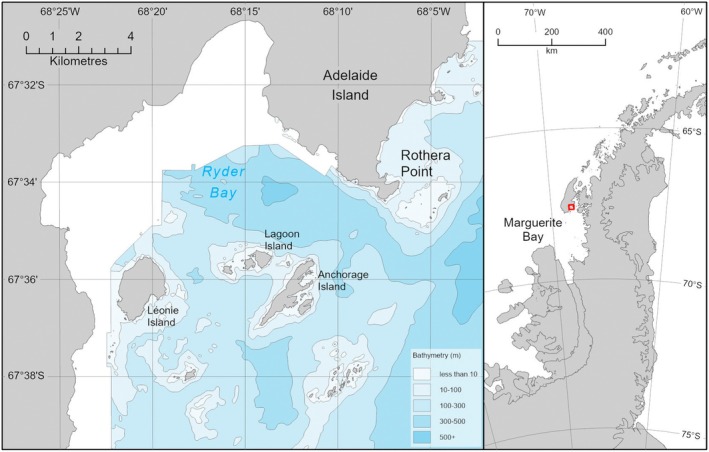
Study location. Map of Ryder Bay. Rothera research station (British Antarctic Survey) is located on Rothera Point. Depth contours are 100 m and the blank areas are unsurveyed. The location of Ryder Bay in relation to Marguerite Bay and the Antarctic Peninsula is shown by the red symbol on the right panel.

The study was based in Ryder Bay on the south‐eastern side of Adelaide Island, and at the south‐western end of Laubeuf Fjord (Figure 1). It has a maximum depth of just over 400 m and opens to the south into northern Marguerite Bay, the largest embayment on the western Antarctic Peninsula. The British Antarctic Survey facility at Rothera Point (67.5689° S, 68.1247° W) was opened in 1975 and has been the site of marine biological and oceanographic research since 1997.

### 2.2 Species

The species included in the study were Common/Antarctic Minke Whale *Balaenoptera acutorostrata/bonaerensis*, Humpback Whale *Megaptera novaeangliae* , Common Killer Whale *Orcinus orca* , Antarctic Fur Seal *Arctocephalus gazella* , Leopard Seal *Hydrurga leptonyx* , Weddell Seal *Leptonychotes weddellii* , Crabeater Seal *Lobodon carcinophaga* , and Southern Elephant Seal *Mirounga leonina* (taxonomy and colloquial names follow the Mammal Diversity Database: https://www.mammaldiversity.org). These are the commoner inshore components of the marine mammal fauna of Marguerite Bay, and with the exception of distinguishing the two species of Minke Whale, are readily identifiable by non‐experts. Occasional records of Sei Whale, *Balaenoptera borealis* , were not included in the study as this species can be identified with confidence only by experienced observers.

Since the 1980s it has been recognised that there are two species of Minke Whale in the Southern Ocean, Antarctic Minke Whale, *Balaenoptera bonaerensis* and an unnamed subspecies of Common Minke Whale, *B. acutorostrata* , referred to colloquially as ‘Dwarf Minke Whale’ (Best 1985). These two species can be difficult to distinguish unless close views are obtained (Risch et al. 2019). While Dwarf Minke Whales are believed to be less frequent south of 60° S (Branch and Butterworth 2001), the two species are sympatric over much of the Southern Ocean (Acevedo et al. 2011). Thiele et al. (2004) recorded Antarctic Minke Whales and unidentified Minke Whale species in the Marguerite Bay area, whereas Friedlaender et al. (2006) reported all of their sightings as *B. acutorostrata*. A small number (~4) of individuals in Ryder Bay that were seen closely (by the senior author) were believed to be Antarctic Minke Whales, based on views of the flipper colouration. However given the uncertainty of the identity of the species to be found in Marguerite Bay, all sightings in this study were referred to Common/Antarctic Minke Whale, and for brevity are referred to hereafter as ‘Minke Whale’.

Along the western Antarctic Peninsula Killer Whales comprise three phenotypically, genetically and culturally distinct ecotypes (Types B1, B2 and A: Pitman and Ensor 2003; Durban et al. 2017 and references therein). The form in Marguerite Bay is type B1 (the Large Pack Ice Killer Whale) which specialises on hunting seals on ice floes (Pitman and Durban 2012).

### 2.3 Marine Mammal Observations

Observations of marine mammals were undertaken as part of the Rothera Time Series (RaTS), a year‐round programme of oceanographic and biological sampling of the Antarctic marine environment (Clarke et al. 2008). Observers included station scientists on oceanographic sampling trips, or undertaking a formal seal‐watch prior to SCUBA diving operations, together with interested station personnel; all were able to identify the study species reliably. Observations were made either from land (0–20 m above sea level) or from small boats undertaking oceanographic sampling. Sightings were generally made with the unaided eye, but binoculars were often used for formal seal‐watches. Sightings were made both opportunistically, while completing other tasks, or from a dedicated weekly transect walk around Rothera Point. The basic data comprising a valid record were date, species and count. The count was the number of individuals visible within the bay at any one time; it is not necessarily a measure of group size as no distinction was made between individuals seen close together or those dispersed across the bay. In addition, observers recorded any notable behaviour or other features of interest in free text. Occasional observations from high elevation (aircraft or nearby mountains) were noted but excluded from the main analysis because of the different likelihood of detection.

From January 1998 to December 2014 sightings of marine mammals were recorded whenever made and collated in monthly spreadsheets for upload to UK. Other scientific demands meant that observations were not possible every day and the median number of days per month with records was 17; these data are referred to as ‘quasi‐daily’. From January 2015 to December 2023 the recording system was simplified, with observations summarised on a weekly basis and the maximum number of individuals seen that week recorded; these data are referred to as ‘weekly’. Over the full observing period (1998–2023) the median number of weeks per year with data was 48 (range 19–52) and the total number of days with observations was 3877, an average of ~150 days each year.

An animal can be recorded as present only if it is seen, but marine mammals vary in their detectability: Killer Whales are generally obvious when present in Ryder Bay, Weddell and Crabeater Seals are visible when hauled out on ice but more difficult to see when in the water, and Southern Elephant Seals are rarely recorded away from land because they spend most of their time underwater and are inconspicuous when at the surface. A record with no marine mammal sightings might represent an absence of whales and seals, undetected presence, or a period with no observations. A day with no whale or seal sightings but records of birds was assumed to be a true zero (observations were made but no marine mammals were seen), whereas a day with no records of either birds or marine mammals was coded as a missing value (no observations on that date).

Detectability is also affected by observing conditions and the key factors affecting the recording of marine mammals in Ryder Bay are weather and ice. Strong winds, blowing snow or fog, conditions that are not infrequent at Rothera, can make detecting marine mammals at sea almost impossible. Weather can also influence the haul‐out behaviour of seals (Aspinwall et al. 2024), and hence their detectability.

**FIGURE 2:**
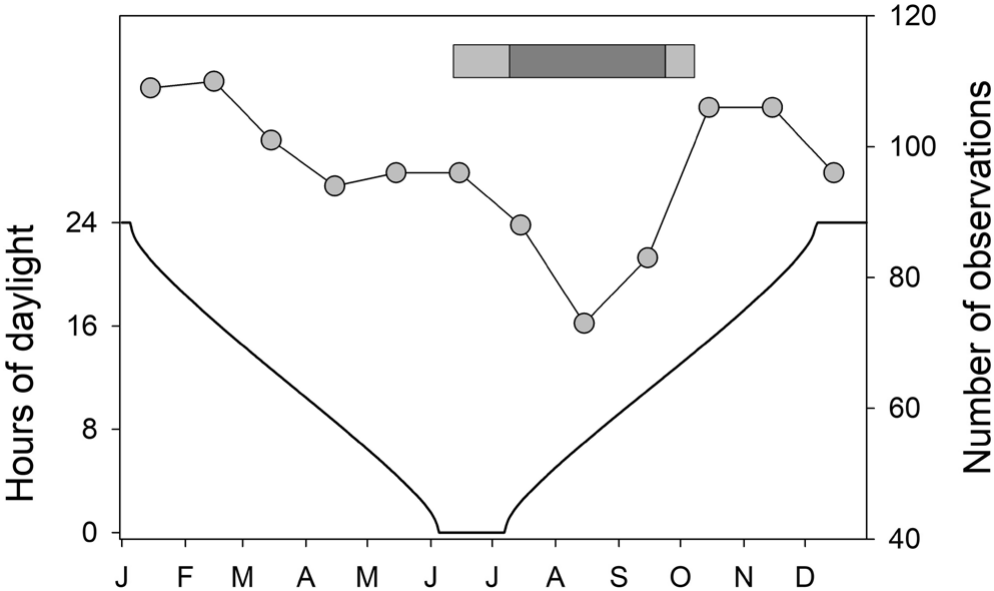
The total number of days with observations of marine mammals for each month of the study period (1998–2023) (line and symbols: Data are plotted at the mid‐point of the month). Also shown are the annual cycle of daylight length (hours) at Rothera Station (solid line) and the period of mean incidence of fast‐ice over the study period (bar), with complete cover (ten tenths) shown in dark grey and partial cover (four to nine tenths) in light grey.

Ryder Bay experiences complete darkness from 5 June to 7 July. The seasonal pattern of monthly records shows that, perhaps surprisingly, these were relatively unaffected by the light climate (Figure 2). The 3 months with the lowest number of observations were July, August and September, which correspond exactly with the period when fast‐ice is most likely to be present (see below). When fast ice coverage is complete marine mammals are absent (or not observed), although when leads or breathing holes are present, they can concentrate otherwise elusive species (Hobson and Martin 1996).

### 2.4 Ice Observations

The RaTS project includes daily observations of sea‐ice. Ice was classified as fast ice, pack ice or brash ice (Meteorological Office 1977). *Fast ice* (*land‐fast ice* in the older literature) is sea‐ice that forms and remains fast along the coast, where it is attached to the shore, to an ice‐front, or between shoals. In Ryder Bay winter fast ice may extend across the entire bay, but disappears during the summer. Fast ice may lock large icebergs in position, preventing them from moving until the fast ice melts. *Pack ice* is any form of floating ice more than 2 m across; in this study no distinction was made between single‐year and multi‐year pack ice. Pack ice floes can move rapidly under the influence of surface currents and wind, and because of this was often referred to by early explorers, mariners and oceanographers as *drift ice*. *Brash ice* is floating ice less than 2 m in size, and is usually the wreckage of larger forms of ice.

The area coverage of each type of ice was scored from 0 (no ice present) to 10 (complete coverage of Ryder Bay). The extent to which pack ice covers the surface is generally greatest near to land, and lessens towards the open ocean so the ice score represents an average for all of Ryder Bay. The region where pack ice coverage is between 15% and 80% is termed the *marginal ice zone* (MIZ) (Dumont 2022); it marks the transition from dense surface ice to open water, and is an important feeding area for many seabirds and marine mammals.

### 2.5 Analysis

**FIGURE 3:**
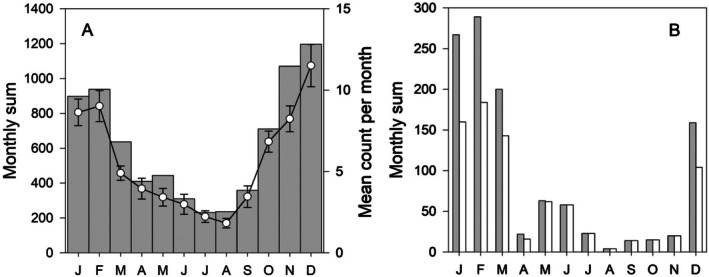
Measures of sighting frequency. A: Seasonal variation in the sighting frequency of Weddell Seals, plotted as the maximum number observed in a given week pooled by month, with the monthly data then summed over the complete study period (1998–2023) (grey bars) and the mean maximum count (±SE) for that month (line and symbol plot) over the study period. B: Seasonal variation in the sighting frequency of Killer Whales, plotted as the weekly maxima summed by month and across all years (grey bars) and as monthly maxima summed across all years (white bars).

Data collected casually and in an unstructured manner can cause problems for subsequent statistical analysis and for this study the challenge was to ensure comparability between the two periods of recording. For the data collected quasi‐daily (Jan 1998–Dec 2014) the maximum count for each species in each week was determined. Data for days 365 and 366 (in leap years) were assigned to week 52. Analysis of these quasi‐daily data showed a strong visual correlation of the maximum weekly count with both the mean number observed that week and the total sum of counts for that week (Pearson correlation, all *p* < 0.05; Figure 3A shows an example for Weddell Seals). For observations collated weekly (Jan 2015–Dec 2023) the maximum count that week was recorded. Initial plots of weekly maxima proved to be noisy, whereas summing the data by month gave a smoother picture. Data were summed rather than calculating a mean because of the statistical behaviour of extreme values (Jenkinson 1955), though direct comparison (Figure 3A) indicates that the impact of this on patterns of seasonality is small.

For visualisation and statistical analysis the weekly maxima were summed by month and the monthly data then summed across all years over the recording period (1998–2023). For those weeks which spanned 2 months, the week was assigned to the month with the larger number of days in that week. In addition for a simpler analysis the monthly maxima were summed across all years. These two data series gave similar seasonal patterns though the seasonality was more marked in the weekly series summed by month (Figure 3B shows an example for Killer Whale); the weekly data summed by month were used for the analyses and plots.

To enable comparison with the marine mammal observation data, the maximum scores for each type of ice were calculated for each week of the study period. In addition, incidence of each ice type was recorded as 0 (absent) or 1 (present), and then averaged over the data series (1997–2023) to determine mean incidence per day, week or month.

### 2.6 Correcting for Ice Presence

**FIGURE 4:**
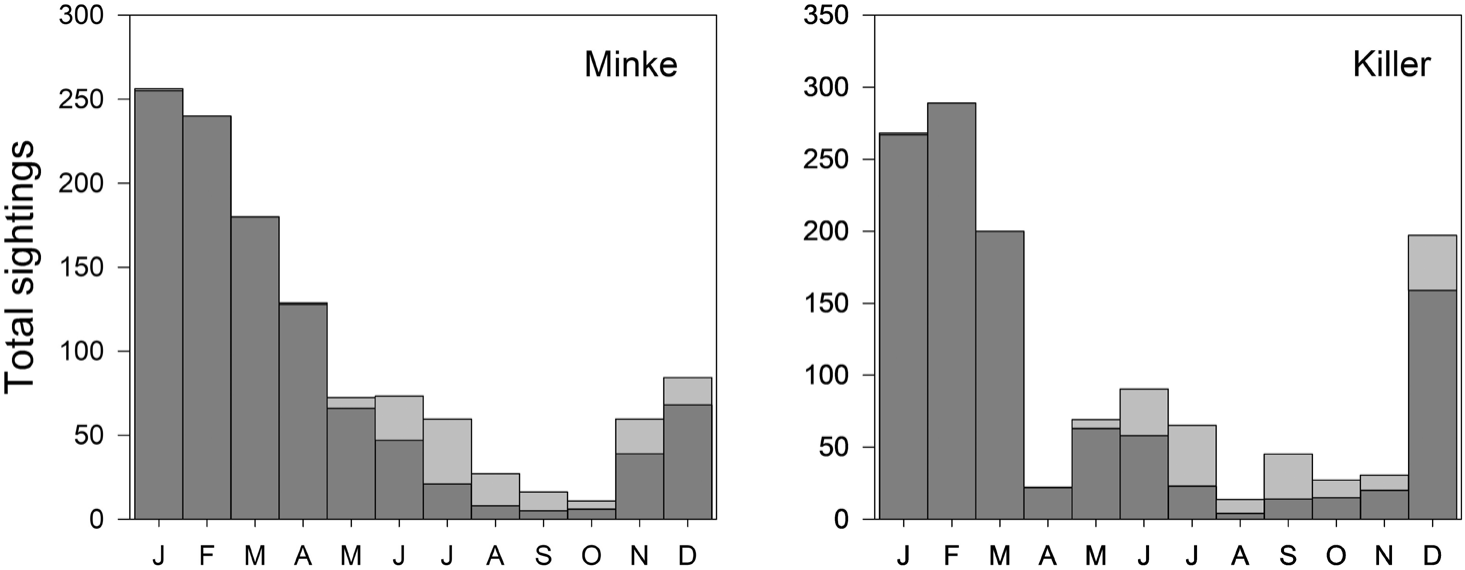
Plots of seasonal sightings of Minke Whales and Killer Whales showing uncorrected sightings data (dark grey) with correction for days with fast‐ice cover (light grey). Data are the maximum number observed in a given week pooled by month, with the monthly data then summed over the study period (1998–2023).

In those species present year‐round in Ryder Bay, the frequency of sightings in winter will be affected by the presence of fast ice: when the sea is covered by fast‐ice, whales will not be detected even if present. To evaluate the extent to which the presence of fast‐ice influenced the observed seasonal pattern of sightings, data were corrected for the number of days in each month with open water. For this analysis open water was defined as when fast‐ice extent was less than four‐tenths coverage (typically this means fast‐ice close to shore but with open water beyond where marine mammals could be seen if present). These corrections were found not to exert a significant influence on the seasonal pattern of sightings (Figure 4 shows two examples).

### 2.7 Statistical Analysis

All analyses were undertaken using R version 4.4.2 (R Core Team 2024). Statistical summaries of the data were obtained with the *pastecs* library and visualised using *ggplot2*, linear models (least‐squares linear regression) were fitted with *glm* in the base R package, and generalised additive models were fitted using *gam* from the *mgcv* library. All models used Poisson error structure because the data were counts, and the *link* function was *identity*.

## 3 Results

### 3.1 Seasonality

**FIGURE 5:**
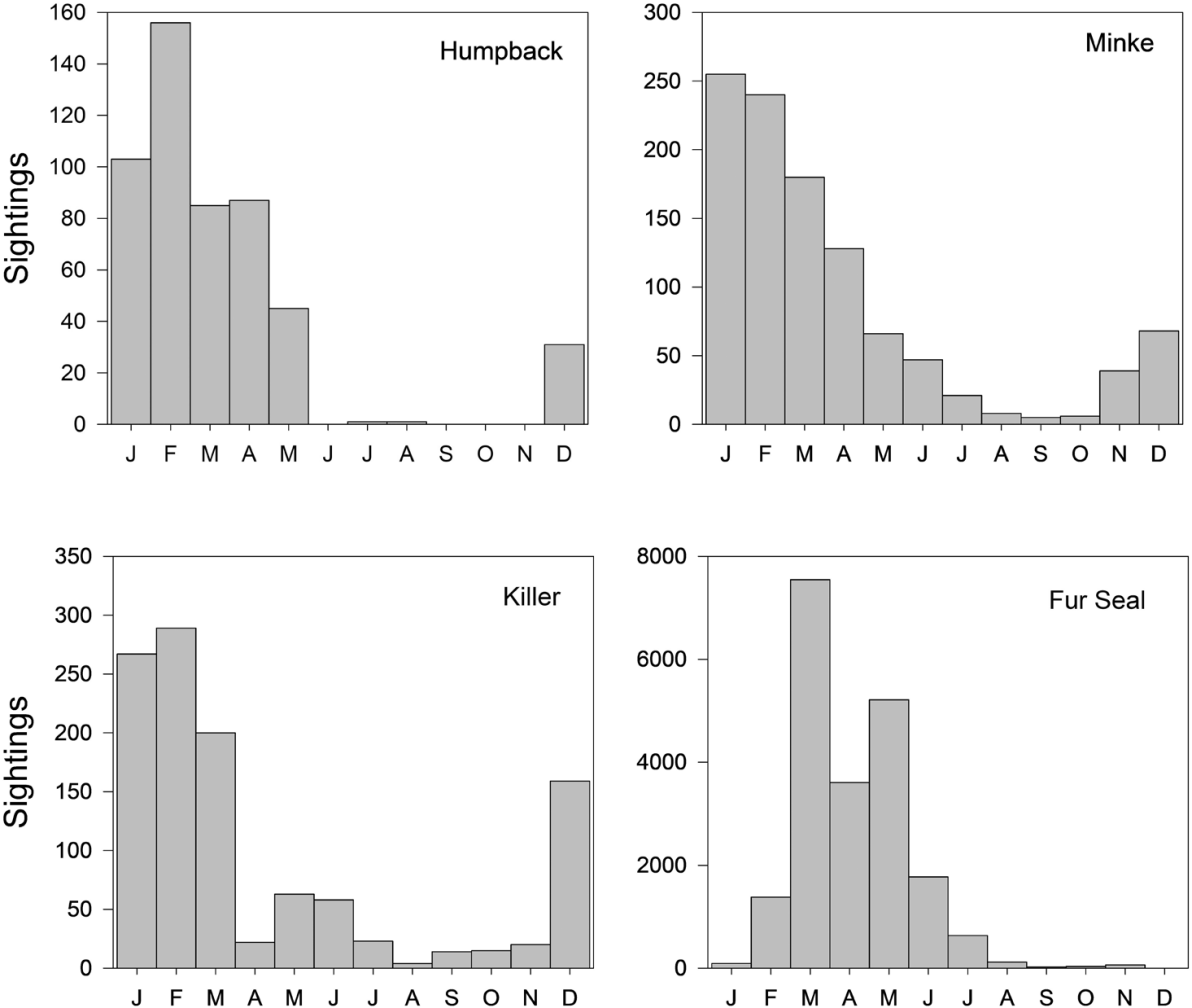
Seasonal occurrence of Humpback Whales, Minke Whales, Killer Whales, and Antarctic Fur Seals in Ryder Bay. Data are the maximum number observed in a given week pooled by month, with the monthly data then summed over the study period (1998–2023).

Humpback Whales are seasonal visitors to Marguerite Bay. The first Humpbacks could be seen in Ryder Bay in any month from December to February, with the peak sighting rate in February and departure in May (Figure 5). They were generally seen feeding in the deeper parts of the bay, and rarely came close to shore. The median maximum daily count was 2 but the largest was 20 and there were seven records of 10 or more individuals seen in the bay at one time. Single Humpback Whales were seen in Ryder Bay during the winter of 2013 on four dates between 13 Jun and 30 Nov, presumably same individual on each date.

Minke Whales are present in the Southern Ocean year‐round (Åsvestad et al. 2024) and were observed in Ryder Bay in all months of the year, though most sightings were in the austral summer (November–April: Figure 5). In Ryder Bay Minke Whales were mostly recorded as singletons, though the median count was 2; the maximum count was estimated at 50 and there were 20 records of 10 or more individuals. As with Humpbacks this species was usually seen in the deeper parts of the bay, and rarely approached the shore.

Killer Whales were seen in Ryder Bay year‐round, though sightings were less frequent in late winter following a distinct secondary peak in sightings during May and June (Figure 5). Killer Whales live in social groups and the median maximum daily count was 5, with an estimated maximum count of 40. Killer Whale pods were frequently seen hunting seals on ice‐floes, spy‐hopping to check for the presence of a seal, and its identity. This behaviour could bring them close to shore, and it was not unusual to see Killer Whales passing right beside the wharf at Rothera research station. On occasion they also swam close to inflatables engaged in oceanographic sampling, even spy‐hopping to (presumably) examine the occupants.

Antarctic Fur Seals are highly seasonal visitors to Ryder Bay, typically arriving in January with numbers peaking in March and most having disappeared by July (Figure 5). Antarctic Fur Seals could be numerous in Ryder Bay, with 122 records of 100 individuals or more, and an estimated peak of 715 animals present in May 2004. There was a significant relationship between the date of the last sighting and the onset of complete fast ice cover (least‐squares linear regression, *F* = 10.18 for 1, 23 df, *p* = 0.004), with a mean time difference of ~20 days.

**FIGURE 6:**
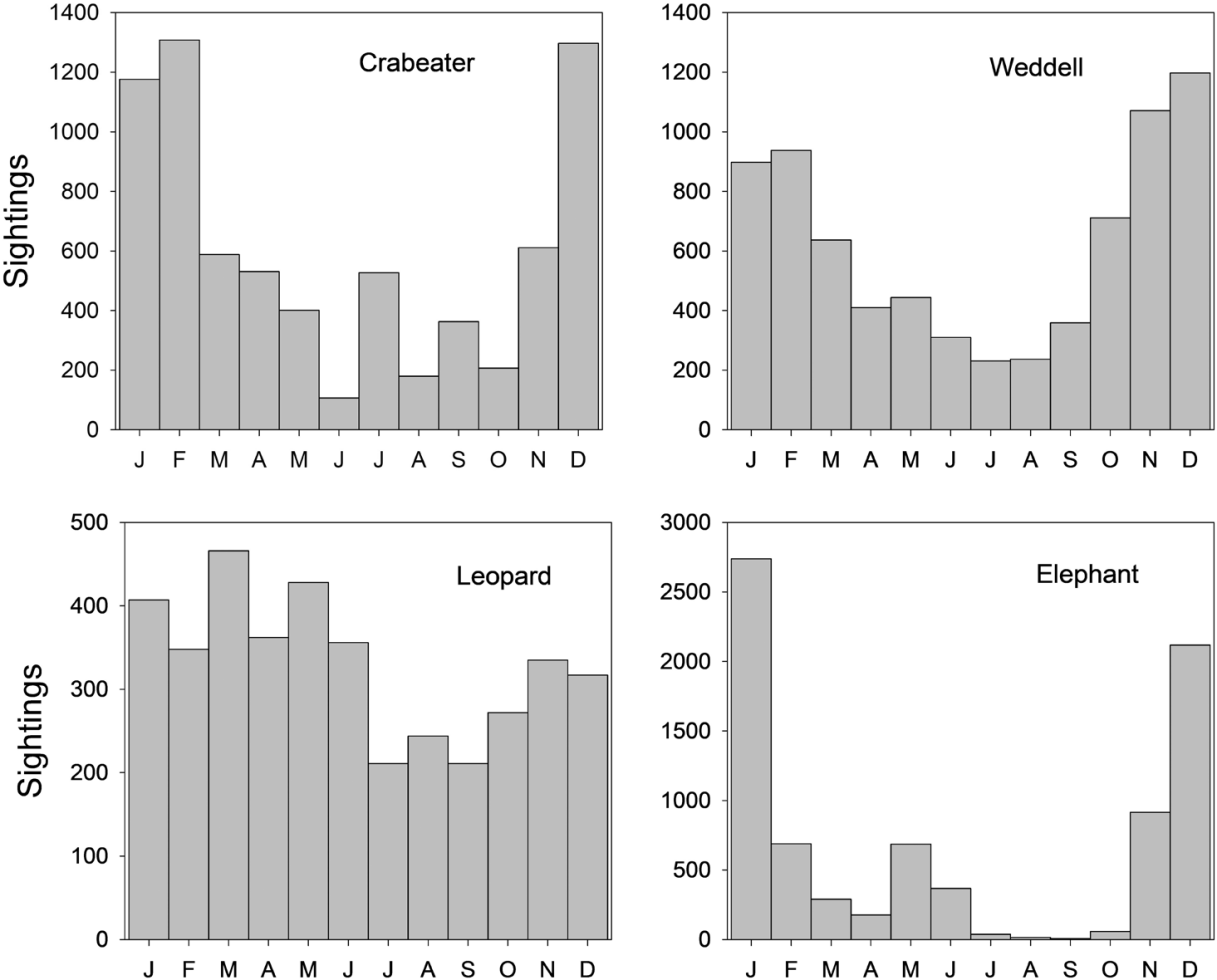
Seasonal occurrence of Crabeater Seals, Weddell Seals, Leopard Seals, and Southern Elephant Seals in Ryder Bay. Presentation as for Figure 5.

Both Crabeater and Weddell seals were present in Ryder Bay all year, although in both cases sightings were more frequent in summer (Figure 6). Crabeater Seals were generally seen in small numbers, hauled out on ice floes, and the median maximum daily count over the observing period was 3. On occasion, however, larger numbers were seen in the open water, and there were two records of ~200 seals present in Ryder Bay (September 2003, April 2010), 8 records with over 100 seals and 29 records of 50 or more. There was a marked seasonality in the frequency of sighting, with most being seen in the second half of summer, after the breeding season, before sighting rate fell away again in winter. Weddell Seals were also generally seen in small numbers, with a median maximum daily count of 4. The peak count was ~100 (Dec 2002) with 4 records of 50 or more seals and 93 records of 20 or more. The seasonal difference in sighting rate was less marked in Weddell Seals than in Crabeater Seals. Occasionally females give birth to pups on remnant fast‐ice attached to the smaller islands in Ryder Bay; pup numbers ranged from 1 (1998) to 12 (2005).

Leopard Seals were the least common of the regularly sighted seal species in Ryder Bay though small numbers were seen all year (Figure 6). The seasonal variation in sighting rate was small, though there was an indication that seals were seen less frequently in winter (July to September). Leopard Seals are typically solitary animals and the median maximum daily count was 1. On occasions, however, there were small influxes into Ryder Bay and there were five records of 5+ seals being seen in the bay; the largest daily count was 11 (September 2021). Leopard Seals were usually seen hauled out on ice floes, but were also often seen hunting close to shore.

Southern Elephant Seals were present in Ryder Bay in summer (Figure 6), with a small additional peak in sightings in early winter (May and June). The period of peak sightings coincided with the main period of moult, during which period Southern Elephant Seals haul out on land. A few pups were seen in some years at Lagoon Island (2 in 2009, 1 in 2013, 2 in 2021 and 6 in 2022), indicating that small numbers of Southern Elephant Seals occasionally breed this far south.

### 3.2 The Influence of Ice

In winter nearshore areas of Marguerite Bay are typically covered by fast ice, though the distance this extends from shore varies from year to year. Beyond the edge of the fast ice is a zone of pack ice, which tends to be densest nearshore, with coverage decreasing towards the open ocean. This pack ice zone is a highly dynamic environment, as floes move in response to surface currents and wind. Because of this mobility, in summer nearshore embayments such as Marguerite Bay can switch rapidly between open water and being covered with light or dense pack ice.

**FIGURE 7:**
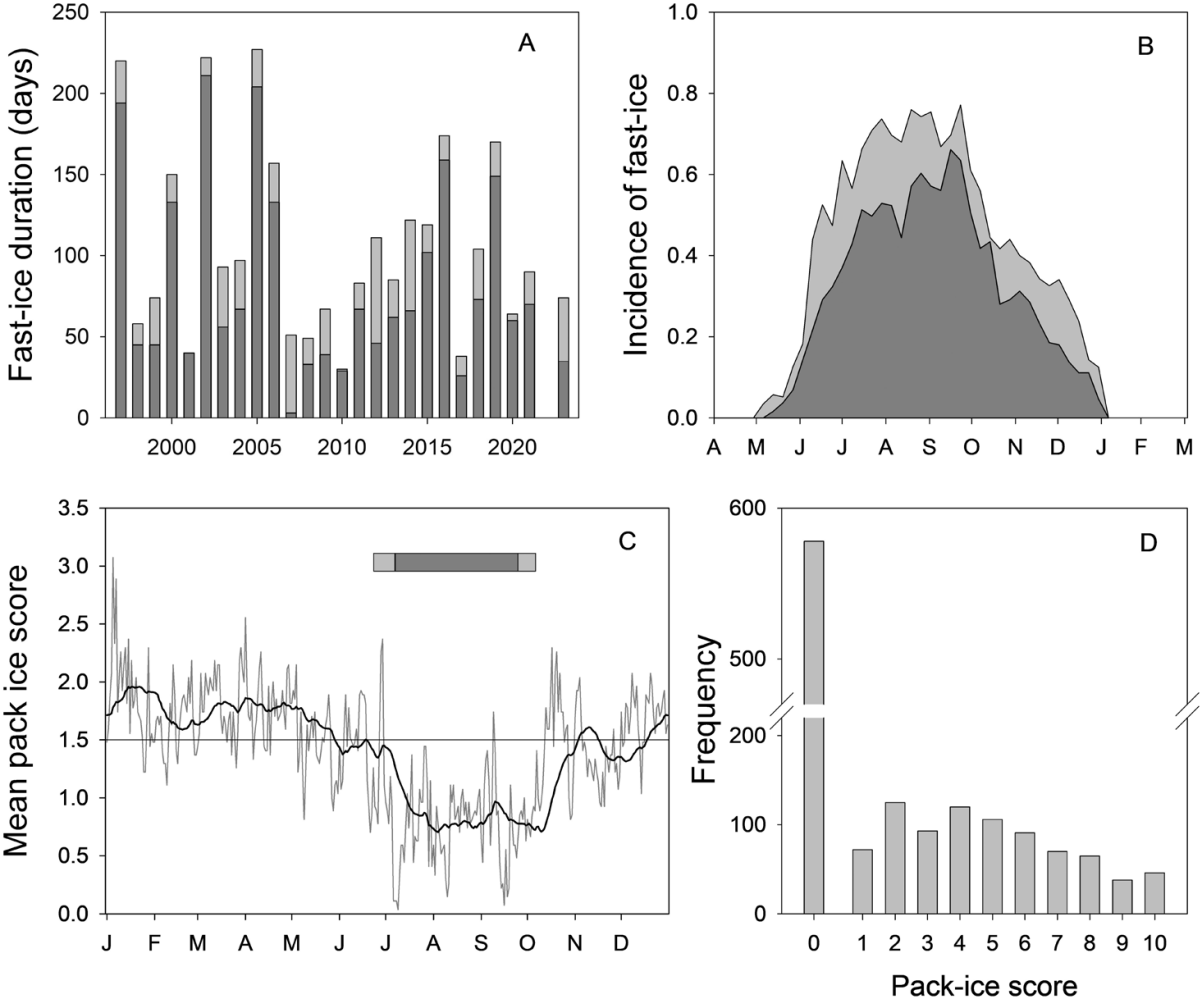
(A) Annual duration of fast ice for 1997–2023. Complete coverage (ten‐tenths) shown in dark grey, and partial coverage (four tenths or more) in light grey. Note the complete absence of fast ice in 2022. (B) Mean incidence of full (dark grey) or partial (light grey) fast ice coverage per week. Incidence score for any one week is either 1 (fast ice is present and coverage exceeds the area threshold) or 0 (no fast‐ice present, or present but below area threshold), and the weekly incidence was averaged over the period 1997–2023. Note that the abscissa runs from April to March, so that the austral winter falls in the centre of the plot. (C) Mean daily pack ice score (coverage in tenths), averaged over the period 1997–2023. The grey line shows the mean daily score and the black line a 30‐day running mean (untapered). The horizontal line shows the lower boundary defining the marginal ice zone (15% coverage, 1.5 tenths). The horizontal bars show the period when the incidence of fast ice exceeds 0.5 when averaged over the same period (light grey: Fast ice score 4–9, dark grey: Fast ice score 10). (D) Frequency histogram of pack ice score (coverage in tenths). The frequency unit is the number of weeks with a given maximum pack ice score. Note that these plots include data for 1997, the year before observations of marine mammals started.

Fast ice formed over Ryder Bay in most winters, though its duration and extent varied greatly from year to year (Figure 7A). Over the period of this study, the duration of complete fast ice cover ranged from 0 days (no fast ice formed at all in 2022) to > 200 days (2002). Fast ice may start forming as early as May and in most years it was typically present from July to October (Figure 7B). In some years fast ice was present continually for long periods, in others it formed and broke up intermittently through the winter.

Pack ice can be present in Ryder Bay in any month of the year (Figure 7C). The apparent dip in the incidence of pack ice in winter is artefactual, as it coincides with the period when fast ice is most likely to be present. The most frequent daily pack ice score was 0 (no ice present, 56% of days, 1997–2023) and also when averaged by week (41% of weeks). When pack ice was present, the most frequent weekly mean score and weekly maximum score were both in the range 2–6 (Figure 7D). These data indicate that Ryder Bay (and by extension wider Marguerite Bay) can be regarded as being within the marginal ice zone in summer (Dumont 2022), but dominated by fast ice in winter.

**FIGURE 8:**
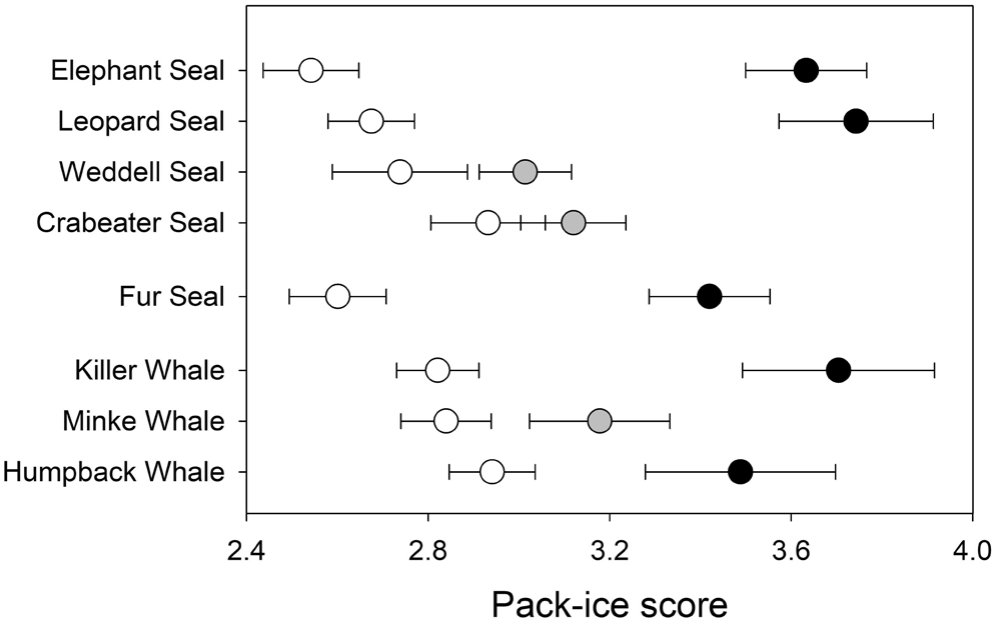
Comparison of pack ice score (mean ± SE) when a species is absent (white symbols) or present. Presence is shown in black when the difference between these scores is significant (*p* < 0.05) and grey when the difference is not significant (*p* > 0.05) (Table 1).

All of the species in this study were seen under all pack ice conditions, from open water to dense pack ice cover. In every case, however, the mean pack ice score when the species was present was higher than when the species was not seen (Figure 8), although this difference was not statistically significant for Weddell and Crabeater Seals, or Minke Whale (Table 1).

**TABLE 1:** One‐way analysis of variance, mean weekly maximum pack‐ice score versus binary factor for species presence/absence.

Species	*F*	df	*p* (two‐tailed)
Humpback Whale	4.57	1, 1246	0.033
Minke Whale species	3.11	1, 1350	0.078
Killer Whale	11.74	1, 1350	0.001
Antarctic Fur Seal	23.16	1, 1350	< 0.001
Crabeater Seal	1.20	1, 1299	0.273
Weddell Seal	2.33	1, 1350	0.127
Leopard Seal	29.77	1, 1350	< 0.001
Southern Elephant Seal	39.64	1, 1350	< 0.001

Abbreviations: df, degrees of freedom; *F*, variance ratio; *p*, probability (two‐tailed).

### 3.3 Long‐Term Change in Sighting Frequency

**FIGURE 9:**
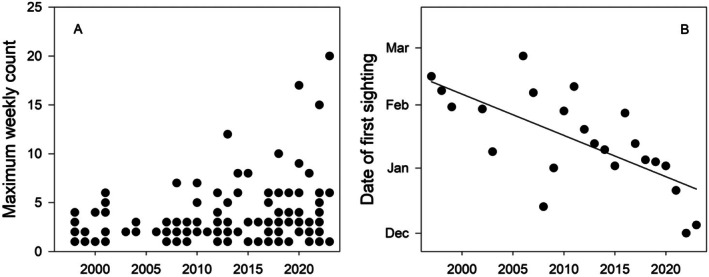
Changes in the occurrence of Humpback Whales in Ryder Bay over the study period (1998–2023). (A) The maximum number of individuals recorded in Ryder Bay at any one time. (B) The date of the first sighting of Humpback Whales in summer as a function of the calendar year. The fitted line is a least‐squares linear regression (Poisson error).

The clearest indication of long‐term change in the sighting frequency of marine mammals in Ryder Bay came from Humpback Whales, where over the period 1998–2023 the number of weeks in the year with sightings has increased significantly (least‐squares linear regression, *F* = 20.3 for 1, 24 df, *p* = 0.001) and the maximum number of individuals seen in the bay at any one time has increased from four in 1998 to 20 in 2023 (Figure 9a). This trend has continued and in Jan 2026 there were at least 30, and possibly up to 40, Humpbacks in Ryder Bay. At the same time, there has been a marked change in the date on which the first returning Humpback Whale is seen (Figure 9b). A simple linear model (arrival date as a function of year) suggests an earlier arrival date by ~21 days (±5, SE) per decade (Table 2). On average the first Humpback Whales arrive in Ryder Bay ~100 days (±6.8, SE) after the local fast ice breaks out and a more complex linear model including this effect of ice suggests a long‐term change in arrival date of ~18 days per decade (Table 2). The date of the last sighting of Humpbacks in Ryder Bay has slipped slightly later over the period of the study. When fitted with a simple linear model (last date as a function of year) the effect size, equivalent to ~21 days per decade, is similar to the change in arrival date but the large variance means that the *F*‐ratio has 0.10 > *p* > 0.05 (Table 2). Including the date of fast‐ice formation reduces the effect size to ~18 days per decade, but this more complex model does not describe the data well (*p* = 0.20: Table 2). However combining the changes in departure date with the earlier arrival date suggests that the duration of the summer stay by Humpbacks in Ryder Bay has increased significantly, by about 41 days per decade (Table 2).

**TABLE 2:** Statistical analysis of long‐term trend in dates of first and last sighting of Humpback Whale in Ryder Bay. Linear least‐squares regression, Poisson error. IceOut: Date (day number) of fast ice breakout; IceIn: Date (day number) fast ice coverage exceeds four‐tenths.

Model	Effect size	SE	*F*	df	*p*
*Arrival date*					
Year	−2.09	0.47	19.42	1, 23	< 0.001
Year + IceOut	−1.85	0.52	7.60	2, 20	0.004
*Departure date*					
Year	2.08	1.11	3.53	1, 23	0.073
Year + IceIn	1.76	1.31	1.74	2, 20	0.20
*Duration of stay*					
Year	4.16	1.19	12.23	1, 23	0.002

Abbreviations: df, degrees of freedom; *F*, variance ratio; *p*, probability (two‐tailed). SE, standard error of effect size.

**FIGURE 10:**
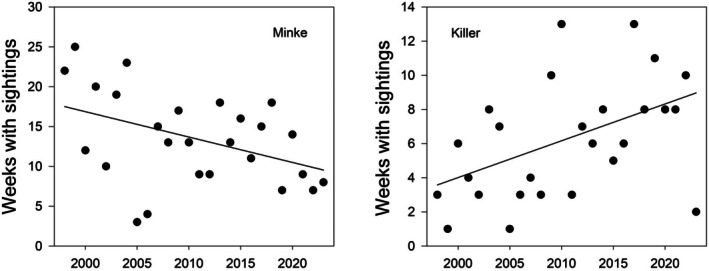
Changes in the occurrence of Minke Whales and Killer Whales in Ryder Bay over the study period (1998–2023). The number of weeks in the year with sightings of Minke Whales has declined (left panel), whereas that of Killer Whales has increased (right panel). The fitted lines are least‐squares linear regressions (Poisson error).

In contrast to the pattern of Humpback Whale sightings, the number of weeks in a year with sightings of Minke Whales has decreased over the observing period by 3.2 (±1.4, SE) weeks per decade and this decline in sighting frequency is statistically significant (*F* = 5.2 for 1, 24 df, *p* = 0.031) (Figure 10). Although there was a small peak in the maximum number of Antarctic Minke Whales seen in the bay in the period 2008–2010, there was no significant overall linear trend in the maximum number seen in any one year (*F* = 1.91 for 1, 24 df, *p* = 0.179).

Killer Whales are present in northern Marguerite Bay all year and the number of weeks with sightings in Ryder Bay has increased significantly over the observing period (Figure 10; *F* = 7.19 for 1, 24 df, *p* = 0.013), but there has been no trend in the maximum number present (*F* = 1.69, for 1, 24 df, *p* = 0.21). In Jan 2026, however, there were three separate groups of Killer Whales in Ryder Bay; one group contained over 15 individuals and the total number in the bay was estimated at 50–60. This is by far the largest number of Killer Whales ever recorded in Ryder Bay since the start of observations in 1998.

**FIGURE 11:**
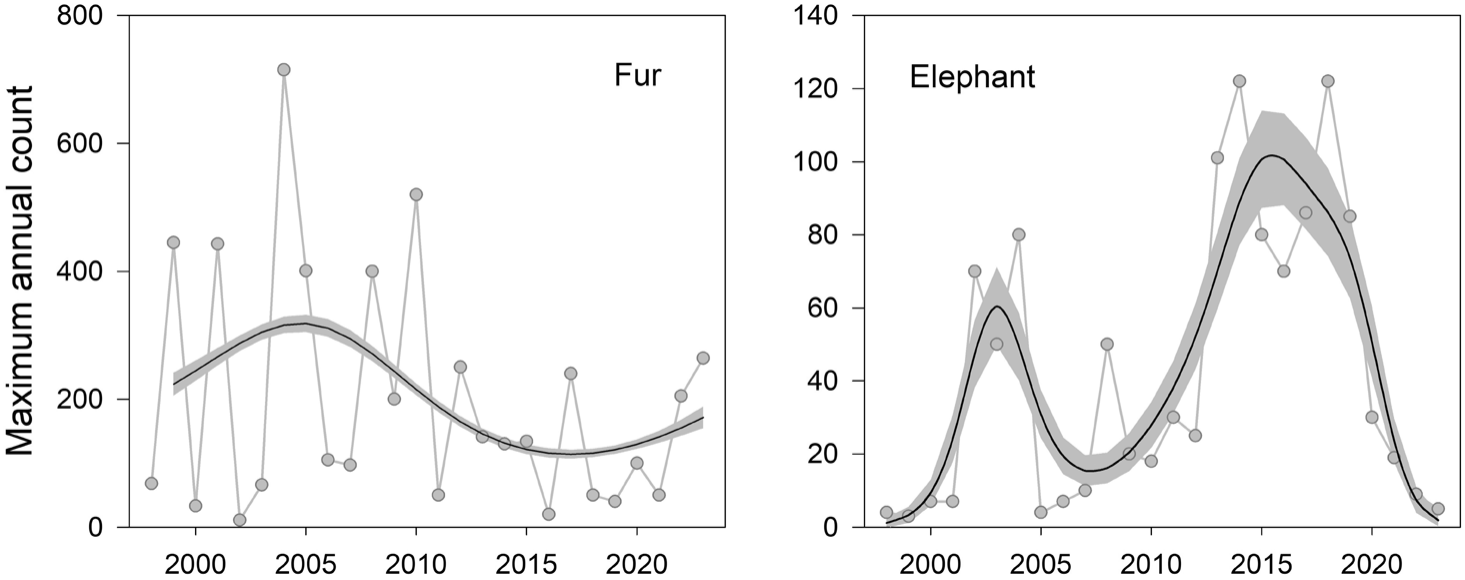
The maximum yearly count of Antarctic Fur Seals and Southern Elephant Seals has varied greatly over the study period (1998–2023). The long‐term trends are generalised additive models with Poisson error, and the 95% confidence intervals shown in grey.

Sightings of the two ice‐obligate seals, Weddell and Crabeater, were both somewhat variable from year to year but neither species showed any long‐term trend in numbers or frequency of sightings (*p* > 0.05 in all cases). In contrast, the maximum annual count of Antarctic Fur Seals at Ryder Bay was highly variable from year to year. There is a visual suggestion of a decline in the very highest counts over the study period, but the overall linear trend is not significant (*F* = 1.69 for 1, 23 df, *p* = 0.21). A generalised additive model (maximum count as a function of year, zero counts excluded) was fitted to provide a simple visual picture of long‐term variability in peak numbers, and this suggests that numbers peaked in the period 2003–2007, and declined thereafter (Figure 11). Southern Elephant Seals showed the most complex temporal pattern in sightings and a generalised additive model suggests distinct peaks in ~2003 and ~2015 with a steep decline thereafter.

## 4 Discussion

Spending most or all of their life at sea, marine mammals have never been easy to study. Early work was confined to periods of haul‐out, occasional strandings or animals harvested for industry. More recently the study of marine mammals in their natural habitat has been revolutionised by the use of passive acoustics (Miller et al. 2015, Calderan et al. 2020; Roca et al. 2023) and/or observation from autonomous aerial vehicles and satellites (Hodgson et al. 2013; Fretwell et al. 2014; Cubaynes et al. 2019; Bamford et al. 2020; Larsen et al. 2025). These developments, while extremely powerful, are often expensive, and some are limited in the temporal and spatial scales they can cover. Under suitable circumstances, simple observational studies still have an important role to play.

While sighting data are valuable, they are not always straightforward to interpret (see for example Martin et al. 2021; Henderson et al. 2023). Sighting frequency does not necessarily reflect population size, even when standardised or corrected for sampling effort, and a seasonal change in sighting frequency, as seen for many species in this study, could simply reflect a change in distribution or behaviour. With these caveats in mind, the observations reported here nevertheless provide valuable insights into the seasonal use by marine mammals of nearshore waters of Marguerite Bay, and long‐term shifts in this use.

### 4.1 Seasonality

Five species of marine mammal are present in Marguerite Bay year‐round (Table 3), though in all cases the frequency of sightings is lower in winter than in summer. These seasonal patterns are relatively unaffected if sighting frequency is corrected for fast ice cover (Figure 4).

**TABLE 3:** Summary of the seasonal presence of marine mammals in Ryder Bay, and phenological changes over the study period (1998–2023).

Species	Seasonal presence
Humpback Whale	Can be present at any time from December to May, though not continuously; occasional individuals in winter. Increase in frequency of sightings and maximum daily count, coupled with increasingly earlier arrival and longer duration of stay
Minke Whale species	Present year round, though fewer sightings August to October. Decrease in frequency of sightings
Killer Whale	Present year round, fewest sightings July to November. Increase in frequency of sightings, but no change in maximum daily count
Antarctic Fur Seal	Present February to July; a few sightings in other months except December. Increase in numbers to ~2005, slow decrease thereafter
Crabeater Seal	Present year round. No detectable trend in sightings or numbers
Weddell Seal	Present year round; a few (up to 12) pups born most years. No detectable change in sightings or numbers
Leopard Seal	Present year round, though slightly fewer sightings in winter. A small increase in the frequency of sightings and maximum numbers
Southern Elephant Seal	Present November to June, hauled out ashore for moulting; occasional sightings July to October; 1–2 pups born in some years. Peaks in maximum numbers ~2003 and ~2015, with steep decline thereafter

Humpback Whales are seasonal visitors to Marguerite Bay, migrating to feed there from the breeding grounds in the Pacific, off the west coast of South America (Modest et al. 2021; Johannessen et al. 2022; Cimino et al. 2023). This population is designated by the International Whaling Commission (IWC) as Stock G (see Seyboth et al. 2023). Sightings of Humpback Whales in Ryder Bay were confined almost entirely to the austral summer and autumn (December to May). The bulk of the population then migrates north to the breeding grounds. It has long been known that a few individuals linger over winter along the western Antarctic Peninsula (Thiele et al. 2004; Johnston et al. 2012) and single individuals, presumably the same individual each time, were seen in Ryder Bay on four occasions in the winter of 2013. More recently, small numbers (up to 12) were seen in Ryder Bay and Laubeuf Fjord in June 2025, suggesting that the number of individuals remaining in Marguerite Bay over winter may be increasing.

Minke Whales are known to be present year‐round in the Southern Ocean, and to favour areas with sea‐ice (Friedlaender et al. 2006; Herr et al. 2019). Minke Whales were seen in Ryder Bay in all months, though sightings were far fewer in winter (Figure 5). Thiele et al. (2004) recorded Minke Whales in Marguerite Bay in winter, noting that sightings were fewer than in summer. This presumably reflects a seasonal difference in distribution and feeding behaviour: Thiele et al. (2004) noted that Minke Whales became more dispersed in winter, with many individuals moving to the marginal ice zone.

Killer Whales were also seen in every month of the year, though markedly less frequently in winter (July to November). It is now known that Killer Whales in the Southern Ocean migrate periodically into warmer waters, where they renew their epidermal tissue. Geolocator data from eight tagged type B1 Killer Whales showed that they left the western Antarctic Peninsula on variable dates in late summer (January to March), travelling rapidly and directly to warmer waters in the South Atlantic, east of South America (Durban and Pitman 2012; Pitman et al. 2020). The winter range of type B1 Killer Whale was described by de Bruyn et al. (2013) as ‘unknown’, but this study indicates that some individuals remain in Marguerite Bay over winter (Figure 5).

Also present in Marguerite Bay during winter are the three ice‐obligate seals (Figure 6). The ecology of both Crabeater and Weddell Seals is tied intimately to ice, but in different ways. Crabeater Seals require pack ice on which rest between foraging bouts (Bengtson and Cameron 2004) and to pup, with the pupping season being September to December. There are no records of Crabeater Seals breeding within Ryder Bay, as breeding is usually deep within extensive areas of pack ice, such as in the Weddell Sea (Wege et al. 2021). In contrast, Weddell Seals use solid fast ice for rearing their pups. This is typically close to land and a recent survey using satellite remote sensing indicated that Weddell Seals breed around the entire Antarctic continent, with the exception of the western Antarctic Peninsula (LaRue et al. 2021). The absence of substantial breeding aggregations of Weddell Seals along the western Antarctic Peninsula presumably reflects the lack of reliable fast ice remaining into the breeding season, although local bathymetry (important for feeding) and distance to land are also important factors (LaRue et al. 2021). However, a few females do give birth in most years in Ryder Bay, with pup numbers ranging from 1 (1998) to 12 (2005). After breeding Weddell Seals move offshore (Beltran et al. 2021) and the frequency of sightings in Ryder Bay drops away. The seasonal variation in sightings of Leopard Seals is far less marked than other species in this study as some individuals of this typically solitary predator remain in nearshore waters year‐round (Kienle et al. 2022).

The two other seal species are seasonal visitors to Marguerite Bay. The main breeding location for Antarctic Fur Seals is South Georgia (Boyd 1993; Forcada et al. 2023) though a small number breed in the South Shetland Islands (Krause et al. 2024). Adult females winter close to the Polar Front (Boyd et al. 1998), whereas males tagged in the South Shetland Islands ranged as far north as South Georgia and as far south as the Bellingshausen Sea (March et al. 2021). The Antarctic Fur Seals in Marguerite Bay are believed to be predominantly sub‐adult males that are not yet ready to breed, as is the case in the South Orkney Islands (Waluda and Dunn 2020). Antarctic Fur Seals typically arrive in Ryder Bay in late summer, but disappear once the fast ice starts to form, a seasonal pattern similar to that seen further north at Pamer Station, Anvers Island (Larsen et al. 2025).

Southern Elephant Seals have a circumpolar breeding distribution on Subantarctic islands, with large populations at South Georgia and Îles Kerguelen (Laborie et al. 2023). Small numbers breed as far south as the South Shetland Islands (Carlini et al. 2003; Negrete et al. 2022; Lanusse et al. 2022). Tracking data shows that Southern Elephant Seals wander widely and are found all around Antarctica although they venture only rarely into the dense pack ice of the Weddell Sea and Ross Sea embayments (Rodríguez et al. 2018). A few pups are born in some years on Lagoon Island in Ryder Bay, which represents a significant southward extension of the known breeding range. However the majority of Southern Elephant Seals in Marguerite Bay are wanderers from more northerly breeding locations and arrive to moult, being present predominantly from November through to June.

### 4.2 Interannual Variability in Fast Ice

The timing and extent of winter fast ice is a key factor in the distribution and ecology of marine mammals (Bester et al. 2016). Knowledge of the dynamics of winter fast ice is thus essential for an understanding of seasonal patterns of marine mammal presence in Marguerite Bay.

The duration of winter fast ice in Ryder Bay was highly variable over the study period (Figure 7). When the duration of fast ice is shorter the winter oceanic mixed layer is deeper; this affects the flux of heat between atmosphere and ocean in the following summer, creating a tendency for years with shorter fast ice duration to cluster (Venables et al. 2013). Thus in 2007 there was a marked reduction in fast ice duration followed by a slow increase, and it took almost a decade before longer periods of winter fast ice recurred. Mariners had long remarked that heavy ice years (when the winter fast ice remained into the spring, affecting the ability of ships to penetrate Marguerite Bay early in the season) tended to come every 7–8 years, and such sub‐decadal variability is clearly evident in the long‐term fast ice record from the South Orkney Islands and also in circumpolar ice dynamics (Murphy et al. 1995, 2014). These dynamics are influenced by wind patterns generated by large scale atmosphere/ocean interactions such as the El Niño Southern Oscillation (ENSO) and the South Annular Mode (SAM) (Murphy et al. 2014).

The pronounced dips in winter fast ice duration in 2007 and 2017, together with its complete absence in 2022 might suggest a significant change in the system, with the potential to affect marine mammal populations. Visually the annual duration of fast ice was often longer in the period 1997–2006 than it has been since, but over the full observing period there was no significant overall (linear) trend in the data (*F* = 0.69 for 1, 24 df, *p* = 0.41). However the oceanographic feedbacks that drive the tendency for years with shorter periods of fast ice to cluster means that a simple linear analysis is compromised by temporal non‐independence. The RaTS ice data series thus emphasises the difficulty in distinguishing decadal‐scale variability from true regime shifts in local fast ice duration, even with relatively long runs of data. The marked drop in fast ice duration in 2017 coincided with a reduction in overall Antarctic sea‐ice coverage (Purich and Doddridge 2023; Himmich et al. 2024; Raphael et al. 2025), suggesting that the variation in fast ice duration in Ryder Bay may reflect wider‐scale drivers. However there are also clear indications that local topography and oceanography exert a powerful influence on nearshore fast ice dynamics (Stammerjohn et al. 2015) and a lack of any long‐term temporal trend was also found for a 28‐year series (1992–2020) of sea‐ice further north at Palmer Station, Anvers Island (Goodell et al. 2024).

This powerful control of nearshore fast ice dynamics by local topography and oceanography makes it difficult to generalise from observations in Ryder Bay to wider geographical areas. However while local ice dynamics will govern whether marine mammals are able to gain access to small bays and inlets, population movements and behaviour will also be responding to ice dynamics on a more regional scale.

### 4.3 Long‐Term Change

The rapid regional climate change along the western Antarctic Peninsula is likely to be having direct effects on marine mammals. The change in sea ice dynamics (Stammerjohn et al. 2008, 2015) may be of particular importance in driving long‐term changes in marine mammals at a regional scale, through changes in access to feeding areas and prey availability. In addition weather patterns (wind, precipitation) are changing with potential impacts on haul‐out behaviour (Aspinwall et al. 2024) and the suitability of nearshore habitats for breeding.

Long‐term sighting data have the potential to detect secular changes in marine mammal populations, but when data are gathered from a single location, as in this study, it is not straightforward to distinguish changes in population size from shifts in distribution or behaviour. The strongest long‐term trend comes from Humpback Whale, where there has been a marked increase in both the frequency of sighting and the maximum daily count (Figure 9a) over the study period (1998–2023). At the same time the maximum number of Humpbacks recorded in Ryder Bay has increased from single figures in the late 1998 to 20 in 2023, and this trend has continued with a group of at least 40 being recorded in January 2026. Interestingly, these recent sightings include observations of breaching and flipper slapping, behaviour which has not been noted in earlier years. The increase in sightings and maximum numbers could suggest that the eastern Pacific population of Humpback Whales is recovering in a similar manner to that well documented for the southwest Atlantic population at South Georgia and the Scotia Sea which is now close to pre‐whaling levels (Bortolotto et al. 2016; Zerbini et al. 2019; Baines et al. 2021; Félix et al. 2021; Jackson et al. 2024).

There is also a clear indication that Humpback Whales are arriving in Marguerite Bay earlier in the season (Figure 9b) and staying longer: over the period of this study (1998–2023) the date that the first individuals have arrived in Ryder Bay has shifted earlier in the season by ~18 days per decade, and the mean duration of the summer stay in Ryder Bay has increased by ~41 days per decade. Analysis of a shorter run of data (2014–2019) from further north at Palmer Station suggested a much faster change in arrival time of ~8 days per year (Cimino et al. 2023) but this trend was not statistically significant and the analysis did not allow for the influence of ice dynamics.

**FIGURE 12:**
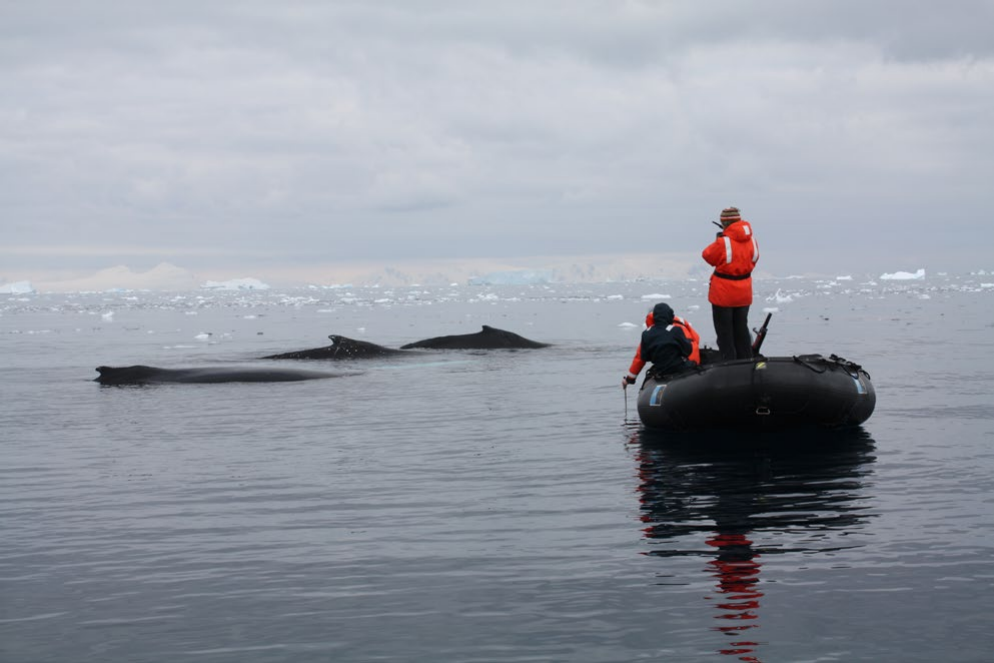
Humpback Whales at the western Antarctic Peninsula. This species appears to be extending its range southwards, arriving earlier on the feeding grounds and departing later, possibly in response to changes in local sea‐ice dynamics. Image: Andrew Clarke.

The increase in sighting frequency and the maximum number of individuals present on any 1 day may indicate an increase in the size of this population, as suggested from mark/recapture models of individuals identified from photographs (Félix et al. 2021; Seyboth et al. 2023), line transect analysis (Branch 2011) and high pregnancy rates in females (Pallin et al. 2018). It may also, however, simply reflect a shift southwards in the population. Recent ship‐based observations suggest that changing oceanographic conditions along the western Antarctic Peninsula may be allowing an increasing number of Humpback Whales to remain and feed further south (Curtice et al. 2015) (Figure 12). While only single individuals were seen in winter in Ryder Bay during this study, and Thiele et al. (2004) recorded only a single Humpback in Marguerite Bay during a winter oceanographic cruise in 2002, more recently in 2025 small numbers (up to 12) were seen in Ryder Bay and nearby Laubeuf Fjord. Taken together these results suggest that the eastern Pacific population of Humpback Whales may be recovering strongly, but is also responding to oceanographic changes associated with regional climate change along the western Antarctic Peninsula. This regional climate change is also affecting penguin populations along the western Antarctic Peninsula.

Like the whales and seals that are the focus of this study, penguins are mobile, long‐lived predators that integrate the effects of environmental variability over large spatial and temporal scales (Fraser and Trivelpiece 1996). Penguin populations have been monitored at various locations along the Antarctic Peninsula since the 1970s. During this time there has been a long‐term decline in the population of the ice‐associated Adélie Penguin *Pygoscelis adeliae* in the Anvers Island region, accompanied by an increase in Chinstrap *P. antarctica* and Gentoo Penguins *P. papua* (Ducklow et al. 2012). The latter two species are relatively recent arrivals to this region: founder populations were only established in 1976 (Chinstrap Penguin) and 1996 (Gentoo Penguin) (Ducklow et al. 2012) and there is no fossil evidence of their presence locally for at least the past 700 years (Emslie et al. 1998). These changes in the Anvers Island region reflect a general southerly movement in the breeding distribution of all three species (Ducklow et al. 2012; Casanovas et al. 2015). In addition to this shift in distribution, the breeding season of these species has advanced by 10–13 days per decade since 2011 (Martinez et al. 2026). These observations suggest that regional climate change along the western Antarctic Peninsula is having wide‐ranging impacts across top predators.

The decrease in the frequency of Minke Whale sightings may be related to the suggested decline in the overall Southern Ocean population (Punt et al. 2014). Based on line transect surveys, the IWC concluded that Antarctic Minke Whales had declined in abundance overall around the Southern Ocean in 2013, although some areas showed increases and some showed declines. In particular there was a large decline in abundance in the area encompassing the western Antarctic Peninsula of 60% between the second (1985/86–1990/91) and third (1992/3–2003/4) circumpolar surveys (International Whaling Commission 2016). However for the decrease in sighting frequency we observed in Ryder Bay, we cannot exclude the possibility that the change simply reflects a shift in distribution, possibly associated with long‐term changes in sea‐ice dynamics.

There has also been an increase in the frequency of sightings of Killer Whales in Ryder Bay (Figure 10). While there was no significant change in the maximum daily count over the study period, very recently (Jan 2026) a total of 50–60 individuals in three groups were seen in Ryder Bay. These results contrast with the overall decline in the western Antarctic Peninsula population of type B1 Killer Whale detected from a mark/recapture model of individuals identified from photographs (Fearnbach et al. 2022). A possible explanation for this difference is that climate change impacts on Type B1 Killer Whales are evident only in the more northerly populations, but at present this is purely speculative.

Of the three ice‐obligate species of phocid seal, neither Crabeater nor Weddell Seals show any indication of long‐term change, though the caveat here is that visual sightings from sea‐level only detect part of the population in the area. Further north at the South Orkney Islands, the breeding population of Weddell Seals have undergone a steep decline following a peak in numbers in the 1980s (Dunn et al. 2025), although there was little subsequent change over the period overlapping with this study at Ryder Bay. There has been a small increase in the sighting frequency and numbers of Leopard Seals, but this could be the result of enhanced observer effort, as this species poses a serious threat to SCUBA operations in Ryder Bay.

The pattern in peak numbers of Antarctic Fur Seals in Ryder Bay (Figure 11) broadly tracks the trend in the population at the main breeding location of South Georgia (Forcada et al. 2023), with a peak in numbers in 2005. Presumably as the South Georgia population decreases in size, fewer non‐breeding males are wandering as far south as Marguerite Bay. Further north at Cape Sheriff, Livingston Island, the small population of breeding Antarctic Fur Seals has declined steadily since 2007 (Krause et al. 2024). These patterns are broadly similar to the trajectory of the population at Bird Island, and by extension the rest of South Georgia (Forcada et al. 2023), suggesting that the long‐term trend in numbers of Antarctic Fur Seals in Ryder Bay reflects the population dynamics at the main breeding location for this species.

The most complex trend was shown by Southern Elephant Seal, where there appear to have been two peaks in numbers, around 2003 and 2015 (Figure 11). A peak in the number of Southern Elephant Seals around 2015 was also noted further north at the South Orkney Islands (Dunn et al. 2025). This species is currently exhibiting differing population trends in the various ocean basins around Antarctica: the population at South Georgia is believed to be fairly stable, those at Îles Kerguelen and Crozet are increasing, whereas the population at Macquarie Island has been declining steadily for many years (Hindell et al. 2017; Laborie et al. 2023). Southern Elephant Seals were, however, impacted severely by the recent outbreak of the Highly Pathogenic Avian Influenza H5N1 virus, and there are likely to be long‐term impacts at all the affected locations (Campagna et al. 2025; Bamford et al. 2025). The most likely explanation for the variability in sighting frequency in Ryder Bay over the study period is that the numbers hauling out to moult are influenced by regional scale variations in the extent and timing of ice (Dunn et al. 2025).

### 4.4 Concluding Remarks

While the observations of marine mammals reported here have been undertaken an opportunistic, unstructured manner rather than as part of a standardised monitoring programme, they have nevertheless contributed valuable insights into the seasonal patterns of occurrence in an important region of Antarctica that was previously little studied. They have also suggested long‐term changes in the population of Humpback Whales, Minke Whales, Antarctic Fur Seals and Southern Elephant Seals along the western Antarctic Peninsula, together with strong evidence for a change in the timing of the seasonal occurrence of Humpback Whales in Marguerite Bay.

The obvious question these observations pose is what the drivers are. Are some marine mammal distributions changing as a direct response to changes in ice dynamics (e.g., by allowing earlier access to, and delayed departure from, more southerly feeding grounds), or to an increase in food availability associated with regional oceanographic changes? Sightings data alone cannot provide an answer to these questions; resolution will require a suite of techniques such as are now being applied to marine mammals at South Georgia and the more northerly sections of the western Antarctic Peninsula, together with continued monitoring of oceanographic changes along the western Antarctic Peninsula, including Marguerite Bay. In the meantime, routine recording of marine mammal sightings in Ryder Bay are continuing as part of the RaTS project, so that future changes in occurrence and timing can be monitored.

## Author Contributions

**Andrew Clarke:** conceptualization (lead), formal analysis (lead), methodology (lead), writing – original draft (lead). **Alysa Fisher:** data curation (lead), formal analysis (supporting), writing – review and editing (supporting). **Hugh J. Venables:** investigation (supporting), writing – review and editing (supporting). **Lucy Allen:** formal analysis (supporting), writing – review and editing (supporting). **Richard G. Davies:** supervision (supporting), writing – review and editing (supporting).

## Funding

This work was supported by the Natural Environment Research Council (NE/R016038/1).

## Conflicts of Interest

The authors declare no conflicts of interest.

## Data Availability

The marine mammal observations reported here and associated ice data are available in the UK Polar Data Centre (https://doi.org/10.5285/a1095421‐60ef‐465c‐8e1d‐1738a9c07e20).
